# Impact of different nutritional approaches on sarcopenia: a protocol for systematic review and network meta-analysis

**DOI:** 10.1186/s13643-023-02215-3

**Published:** 2023-03-13

**Authors:** Jung-Hyun Kim, Sung-Min Kim, Yong-Chan Kim, Byung-Kwan Seo

**Affiliations:** 1 Department of Acupuncture & Moxibustion, Kyung Hee University Hospital, Gangdong, 892, Dongnam-ro, Gangdong-gu, Seoul, 05278 Republic of Korea; 2 Department of Orthopaedic Surgery, Kyung Hee University Hospital, Gangdong, 892, Dongnam-ro, Gangdong-gu, Seoul, 05278 Republic of Korea; 3 Department of Acupuncture & Moxibustion Medicine, Kyung Hee University College of Korean Medicine, Kyung Hee University Hospital at Gangdong, 26, Kyungheedae-ro, Dongdaemun-gu, Seoul, 02447 Republic of Korea

**Keywords:** Nutrients, Sarcopenia, Network meta-analysis, Systematic review, Protocol

## Abstract

Although it is known that proper nutrition is effective in managing sarcopenia, the most powerful nutrients have not yet been determined. This study is designed to investigate the effects of various nutritional approaches on muscle mass, muscle strength, and sarcopenia prevention in systematic reviews. In study design, network and pairwise meta-analyses of randomized clinical trials were considered. Clinical studies regarding the nutritional effects associated with the physiological activity of skeletal muscle and management of sarcopenia will be covered. The main outcomes will cover the following five elements: anti-fatigue impact with skeletal muscle, muscle atrophy prevention, differentiation level with skeletal muscular cell, anti-inflammatory effect, and muscle injury prevention. Authors will conduct the study selection, extracting data process, and methodological quality investigation.

**Systematic review registration**

OSF registry (ethical approval number: https://osf.io/ye4q7).

## Introduction

A progressive loss of muscle function along with muscle mass and strength in skeletal muscles is known to affect the quality of life in older adults [1]. The changes associated with this form of aging can cause a loss of function with muscular protein and age growth and can also be secondary to sarcopenia [2, 3].

Although the rate of aging varies across different populations depending on the level of development, an aging population is common in all regions and countries [4]. The proportion of individuals aged 60 years or more is increasing every year and is estimated to more than double by 2050 [5]. The aging population is expected to have a large and wide effect on all features of society [4].

Sarcopenia can reduce of skeletal muscle mass and weaken muscle strength; thus, the morbidity of sarcopenia puts the elderly at a risk of side effects, such as impairment and unfertile quality of life [6]. The pathological causes of sarcopenia contain reduced bodily activity, lack of nutrition, and growing activity in cytokines [7].

The integration of appropriate nutrients associated with the use of dietary supplements has been first-hand approach to prevent sarcopenia. An increasing amount of clinical evidence has demonstrated that the nutritional intake manages a crucial role in the treatment of sarcopenia. Additionally, nutritional modifications improve the muscle mass in the skeletal area [8, 9] and muscle strength in individuals with settled muscle tension.

Although it is already well known that proper nutrition is effective in patients with sarcopenia, the most powerful nutrients in the management of sarcopenia and improvement in muscle strength have not yet been determined.

To the best of our understanding, limited studies have been developed to examine the efficacy of different nutrients in the treatment of sarcopenia. Moreover, the question which nutritional approach offers the most powerful advantages in improving decreased muscle function remains to be answered.

This article aims to compare the effects of various nutrients on patients with sarcopenia in this network meta-analysis.

## Methods

### Registration

This network meta-analysis with systematic review of articles was pre-listed in the OSF registry (registration DOI:https://doi.org/10.17605/OSF.IO/YE4Q7) and will be reported according to the guides for network meta-analysis protocol (PRISMA-P) [10].

### Eligibility criteria

#### Participants (P)

Human studies engaged in the potential approaches relevant to the skeletal muscle mass or strength for the management of sarcopenia will be selected. Among human studies, subjects aged 18 years or older and of both genders will be included. Eligible subjects should be diagnosed as primary or secondary sarcopenia patients. The following four criteria must be met to be diagnosed with sarcopenia: muscle mass reduction, decreased muscle strength, muscularity assessment in daily life, and reduced walking speed. If patients’ behavior is slow, you will have trouble with walking, climbing, and getting up from the chair. Also, you will possibly have frequent falls. These changes make you proceed with the steps for diagnosis. If patients’ behavior is getting slow, must get help to walk, hard to get up from your chair, climb stairs is overwhelming, or have frequent falls recently, guidelines consider these patterns as a decrease in muscle strength needed for your daily life and proceed with the steps for diagnosis. If a man’s hang grip is measured less than 27 kg or a woman’s hand grip is measured less than 16 kg, it is a muscle loss, and if the five times standing up in the chair takes longer than 15 s, both men and women are diagnosed as a muscle loss. X-ray, MRIs, or bioimpedance analysis (BIA) can be used to diagnose sarcopenia with muscles with extremities. Favorable effects will be investigated with the following five categories: anti-fatigue impact with skeletal muscle, muscle atrophy prevention, differentiation level with skeletal muscular cell, anti-inflammatory effect, and muscle injury prevention [11].

#### Interventions (I)

Regarding the abovementioned inclusion criteria, we will noticeably include one of the following nutrients and a control group or at least two nutrients (multiple control groups).

The suitable types of nutritional alternatives will be included as follows:

- Vitamin D
- Omega-3 fatty acids
- Albumin

The following categories of randomized-controlled trials will be ruled out as follows:

- Any kind of optimized or balanced diet including low-carbohydrate, high-protein, low-fat, and vegetarian diets
- Placebo substance contained in dietary supplements
- Studies with interventions other than nutritional approaches
- Nutrients are partially applied or not applied in all the experimental and control groups.

#### Comparison or comparator (C)

The comparator was classified as control, routine care, and any kind of medications.

#### Outcome measures (O)

Clinically controlled studies of eligible nutrients are usually small, and that distribution of data is difficult to investigate for studies with small sample. Consequently, priority will be settled to the use and analysis of dichotomous variables both for effectiveness and acceptability in this present review.

The main outcomes are interpreted comprehensively in accordance with the following five domains:

1. Anti-inflammation or antioxidant effects: Level of cytokines which is relevant with inflammation (e.g., sTNF-RII, IL-6, TNF-a, IL-1ra, IL-8) or level of antioxidant enzyme (e.g., superoxide dismutase, glutathione peroxidase, glutathione reductase)
2. Muscle impairment prohibition: Enhancement in the recovery of muscle strength and delayed-onset muscle soreness (DOMS).
3. Anti-fatigue outcome: Blood urea nitrogen (BUN) levels or serum lipid profiles used for cardiovascular risk prediction.
4. Muscular atrophy prevention: Risk of mobility impairment, muscular atrophy after severe workout, skeletal muscle mass maintenance, and muscle strength.
5. Muscle reconstruction effects: Enhancement of several body conditions, muscle strength improvement in handgrip, the ratio of plasma follistatin/myostatin, and skeletal muscular mass

Additional outcomes include the following features: (1) Any kind of adverse events (e.g., headache, nausea, indigestion) and (2) the quality of life (e.g., SF-36, Euro-Qol-5 dimensions).

### Study design

The randomized trial comparison will be conducted among possible nutrients with a minimum application timeline of 3 months based on the up-to-date Asian Working Group for Sarcopenia: 2019 Consensus Update on Diet and Sarcopenia Management [12].

### Information sources

The suitable databases and terms for the retrieval process will be discussed by all the relevant researchers. Prior to the literature searching process, terms for the search and target databases are determined after comprehensive discussion. Two independent research workers will conduct the retrieval with online literature, research decision, data extraction, and methodological quality evaluation. The next online database will be investigated for articles from the initiation to the present. The following databases will be considered as data sources: PubMed, Embase via Elsevier, Ovid MEDLINE, Central Register of Control Tests, Web of Science, Scopus, Korean Studies Information Service System, Korean medical database (KMbase), and the Korean search engine.

### Search strategy

The search strategy will integrate the textual terms that define sarcopenia with the descriptions explaining the nutritional options. Preliminary search results showed the largest number of search results in MEDLINE via PubMed. Projected strategy for MEDLINE via PubMed is presented with Table 1. Authors will recheck the reference lists of all the selected trials and related articles. Additionally, authors who are specialized in the clinical domain of sarcopenia will identify publications regarding the interventions for sarcopenia management. We will search for undisclosed work through major conference processes and websites such as ProQuest papers, EThos, and OpenGrey. Furthermore, additional unpublished data will be demanded from the investigators. The National Institute for Health and Care Excellence (NICE, UK) and the Quality and Economics of Intramuscular Healthcare will be scanned for any information that is not already distributed. Chinese databases will not be retrieved to avoid the possible biases that may be contained by selecting trials without further information. Despite numerous randomized controlled trials are distributed in Chinese journals, in many of these articles reported that they do not guarantee proper randomization processes [13]. Instead, researchers will collect all possible studies irrespective of their nations of origin, retrieved in the international databases mentioned above, and meet inclusion/exclusion criteria.

**Table 1:** Search strategy for the MEDLINE via PubMed

Search: #14 AND #16 filters: randomized controlled trial
(("vitamin d"[MeSH Terms] OR "ergocalciferols"[MeSH Terms] OR "fatty acids, omega 3"[MeSH Terms] OR "albumins"[MeSH Terms] OR "proteins"[MeSH Terms] OR "amino acids"[MeSH Terms]) AND "randomized controlled trial"[Publication Type] AND ("sarcopenia"[MeSH Terms] AND "randomized controlled trial"[Publication Type])) AND (randomizedcontrolledtrial [Filter])
Translations
vitamin d [MeSH Terms]: "vitamin d"[MeSH Terms] OR "ergocalciferols"[MeSH Terms] Omega 3 fatty acid [MeSH Terms]: "fatty acids, omega-3"[MeSH Terms] Albumin [MeSH Terms]: "albumins"[MeSH Terms] Protein [MeSH Terms]: "proteins"[MeSH Terms] amino acid [MeSH Terms]: "amino acids"[MeSH Terms] randomizedcontrolledtrial [Filter]: randomized controlled trial [PT] sarcopenia [ MeSH Terms]: "sarcopenia"[MeSH Terms] randomizedcontrolledtrial [Filter]: randomized controlled trial [PT]

### Study records

#### Data management

With a standard structure, the eligibility evaluation with the titles and citations acquired from retrieval will be conducted by two reviewers. The reviewers will not be blinded to authors, journals, or countries. The research form will be tested on a trial basis by the review members. Discrepancies can be resolved by agreement or, if necessary, by discussion with a third researcher. Handling title and abstract, the two researchers will independently verify the eligibility of the full-text papers using the standard forms after a screening of potentially qualified studies. Likewise, if there are any disagreements, the authors will first discuss for consensus. If needed, the third author will intervene with assistance. On the selection of title, abstract, and full manuscript, consistency between the two reviewers will be evaluated by reviewing the raw consistency and the unweighted kappa (k). If the calculated number shows ≤ 0 in kappa score, it will be judged as poor agreement. In contrast, if the score is over 0.80 in kappa score, it will be considered as almost perfect agreement [14].

### Selection and data collection process

The available data will contain the demographic traits of the participants, types of possible nutrients, and all possible outcome measures, data at the baseline, and post-intake data points. If possible, we will first extract the mean or mean difference from the baseline data and standard deviations (SDs). We will additionally explicate target information from which SD could be derived. It could be standard error or confidence interval (CI). In addition, the number of events and the total number of patients per arm or the odds ratio with a measure of uncertainty will be derived. If a trial demonstrates results at more than one time point, the data for all time points will be demonstrated.

### Risk of bias in individual studies

After a pilot trial (*n* = 3) by two independent reviewers (the two reviewers are not blinded to authors) [15], the risk of bias will be assessed. The revised version of Cochrane risk-of-bias tool (RoB 2.0) for randomized controlled trials will be utilized in this procedure [16]. Given the abovementioned domains in the tool, the reviewer followed the algorithms to answer the signaling items and conclude the risk of bias as “low,” “some concerns,” or “high.” Finally, a “risk of inconsistency” summary and a chart will be established to demonstrate the outcomes. Regarding the previous procedure, two independent researchers will investigate the whole assessing process. If a disagreement grows in the process, it will be arbitrated with the third researcher.

### Data synthesis

#### Information flow shown in the network established

The available evidence will be demonstrated in the newly established network diagram. The size of the nodes will show the power of clarity accumulated for each nutrient. Additionally, the breadth of each edge will positively reflect the inverse of the variance of the summary effect of each direct nutritional intake comparison. The color of each edge will show risk of bias (refer to risk-of-bias investigation section).

#### Pairwise meta-analysis

For meta-analysis, we will evaluate the similarity of interventions by different candidate nutrients and interventions due to specific nutrients included in each selected study and whether the same outcomes were covered in the studies in which these interventions were evaluated. To evaluate this, the individual characteristics of RCT included in present study will be summarized and reported. The clinical heterogeneity of the each RCT can be demonstrated by checking the baseline characteristics of patients affected by sarcopenia.

For each pairwise comparison, data will be synthesized to access odd ratio (OR) or standardized mean difference (SMD). For dichotomous data, ORs will be calculated; for continuous data, SMD will be estimated. Statistical heterogeneity across each trial will also be evaluated with the *I*^2^ statistics. Fixed effects model should only be used when if it is predictable that all respective study shares the one same common effect. On the other hand, a random effects model presumes that each study estimates a different underlying true effect, and these effects have a normal distribution. Authors will synthesize SMD or ORs with fixed effects model if *p*-value is ≥ 0.1 and *I*^2^ is ≤ 50%; the random-effects model (REM) will be selected otherwise.

#### Network meta-analysis

WinBUGS originated from MRC Biostatistics Unit will cover the process of network meta-analysis in this research. It aims to draw the combined outcome between two interventions and rank the effectiveness among all possible arms in trials [17]. Originally, WinBUGS can be classified as Bayesian software and established to build complex statistical models with the Markov chain Monte Carlo method. Median rankings will be numbered as point estimations of effectiveness in multiple nutrients to facilitate the practicality. For setting up network meta-analysis, a random effects model with indirect/mixed treatment comparison will be used [18]. Moreover, 95% credible intervals (CIs) will be organized with the 2.5 and 97.5 percentiles acquired through Monte Carlo simulation of 10,000 iterations to identify the significance of nutritional efficacies [19]. We will assess the possible data repeatedly in the reports on several trials. Thus, we can draw direct and indirect estimates independently before beginning a network meta-analysis [20].

#### Transitivity assumption

Transitivity refers to the basic supposition of indirect comparisons and network meta-analyses. Not meeting this assumption conciliates the validity of recommendations from a network of eligible articles. The turnarounds in body weight and mean baseline age will be acknowledged as possible effect modifiers.

#### Confidence in cumulative evidence

To investigate the firmness of extracted evidence, the Grading of Recommendations, Assessment, Development, and Evaluation (GRADE) approach for network meta-analysis will be utilized [21–23]. In conjunction with the risk-of-bias scoring for every outcome, GRADE assessment contains the rating of evidence for indirectness, inconsistency, and dissemination bias. If the network estimate demonstrates high certainty and a similar contribution of direct and indirect evidence, the rating score will be high. In contrast, in case of incoherence and imprecision, the score will be further downgraded. If there is no enough evidence or certainty is rated moderate or low, the indirect estimate will be scored by the lower of two direct comparisons with first-order loops. Also, this could be further underrated for intransitivity.

#### Selection bias assessment

The risk of selection bias is relatively high in controlled trials, in particular with placebo-controlled trials [24]. To judge whether outcomes in imprecise studies differ from those in more precisely designed trials, the comparison-adjusted [25] and contour-enhanced funnel plots [25] will be utilized. Moreover, to detect association’s study size and effect size, network meta-regression model will be considered [26].

### Subgroup analyses

If there is a possibility of heterogeneity or inconsistency, we will investigate the attainable sources via subgroup and meta-regression analyses. Subgroup analyses will be conducted for sarcopenia status, duration of study, sample size, gender, and age. Contour-enhanced funnel plots [27] will be utilized to analyze whether funnel plot asymmetry is likely to be clarified by publication bias. In case publication bias is discovered, we will make an effort to fit a selection model that presented the relationship between the relative effects and anticipation of an article to be distributed, and we will acquire the relative effects adjusted for the effect of the publication bias [28].

## Discussion

The term sarcopenia refers to the condition of declining muscular function and mass, mainly because of aging [29]. Sarcopenia refers to increased risk of fall and debilitated ability to carry out daily livings that often induce disability and decrease independence. Regardless of its clinical importance, sarcopenia is often less considered in the routine clinical domain, partly due to the lack of accessible methods in diagnosis [30]. Generally, the treatment of sarcopenia concentrates mainly on physical therapy or exercise for gait training and enhancing muscle strength [31]. For now, there are no reliable drugs for sarcopenia [32]. With present study, we will investigate clinical trial studies on the effectiveness of nutritional alternatives in relation with the muscular health and management of sarcopenia. In this regard, the following five domains will be examined: anti-inflammatory effect, antioxidant impact, prevention in muscular damage, anti-fatigue effect, the preventive effect on muscular damage, and impact on muscular regeneration. We anticipate that our study will establish the basis and aid in proposing optimized clinical options for health policy makers, practitioners in the clinical domain, patients, and relevant experts. Additionally, we expect that patients with sarcopenia will accept proper nutritional advice from physicians in consonance with clinical evidence.

## Data Availability

The original data and materials used in present study are available in public.

## Abbreviations

BIA: Bioimpedance analysis
BUN: Blood urea nitrogen
CI: Confidence interval
DOMS: Delayed-onset muscle soreness
GRADE: Grading of Recommendations, Assessment, Development, and Evaluation
IL: Interleukin
OR: Odds ratio
SD: Standard deviation
SF-36: 36-Item Short-Form Survey
SMD: Standardized mean difference
TNF: Tumor necrosis factor
